# Long-term outcomes of unilateral lateral rectus recession versus recess-resect for intermittent exotropia of 20-25 prism diopters

**DOI:** 10.1186/1471-2415-14-46

**Published:** 2014-04-08

**Authors:** Hae Jin Kim, Dongwook Kim, Dong Gyu Choi

**Affiliations:** 1 Department of Ophthalmology, College of Medicine, Hallym University, Kangnam Sacred Heart Hospital, Youngdeungpo-gu, Seoul, Korea

**Keywords:** Intermittent exotropia, Unilateral lateral rectus recession, Recess-resect, Moderate-angle exotropia

## Abstract

**Background:**

The purpose of this study was to compare surgical outcomes of unilateral lateral rectus recession (ULR) and unilateral recess-resect (RR) for intermittent exotropia of 20-25 prism diopters (PD).

**Methods:**

In this retrospective study, ULR was performed on 82 patients and RR on 98 patients for the treatment of intermittent exotropia of 20-25 PD with a follow-up period of 24 months or more. The main outcome measures were postoperative exodeviation angles and final success rates. A surgical success was considered to be an alignment within 10 PD.

**Results:**

The mean follow-up duration after the surgery was 53.8 ± 26.4 months in the ULR group and 52.5 ± 27.4 months in the RR group (p = 0.482). The mean deviation angles at postoperative 1 day were -0.49 PD (esodeviation) in the ULR group and -1.98 PD in the RR group. Subsequently, at postoperative 1 week, 1 and 3 months, the deviations became more exotropic in the ULR group than in the RR group (p < 0.05). However, the mean deviation angles at 6 months, 1 and 2 years and at the final follow-up did not significantly differ between the two groups. Surgical success at the final follow-up was achieved for 50 patients (60.9%) in the ULR group and 55 patients (56.1%) in the RR group (p = 0.511).

**Conclusions:**

ULR is an effective surgical method for treatment of moderate-angle intermittent exotropia of 20-25 PD, showing results similar to those of RR.

## Background

The choice of surgical procedure for treatment of intermittent exotropia depends on the type of exotropia and the preoperative exodeviation angles. Burian et al. classified intermittent exotropia based on differences of distance and near deviation, according to which classifications they recommended different surgical methods [1-3]. For the basic type of exotropia, in which the distance deviation is within 10 prism diopters (PD) of near deviation, they recommended recession of the lateral rectus muscle with resection of the ipsilateral medial rectus muscle (RR) [4]. For the divergence excess type of exotropia, in which the exodeviation is at least 10 PD larger at distance than at near, they recommended bilateral lateral rectus recessions (BLR) [4]. Also, Burian and Spivey [4] defined the condition in which distance exodeviation is larger than near, but near deviation increases within 10 PD of the distance deviation after monocular occlusion as the pseudo-divergence excess type. For this type, RR surgery was recommended as a basic type of exotropia. However, the study by Kushner showed that BLR might be equally effective in the pseudo-divergence excess type and the basic type of exotropia [5].

Unilateral lateral rectus recession (ULR) as a treatment for moderate-angle exotropia has been studied and reported since 1950 [6]. The published ULR success rates have varied widely [7-12] depending on the definition of success rate and the follow-up period. Nelson et al. [7] found it to be a safe and effective treatment for small-to-moderate-angle exotropia. Moreover, several authors have reported, for this procedure, a low rate of overcorrection, which lowers the risk of developing amblyopia and the consequent loss of binocular vision [8,13]. Recently, it was reported that ULR can lead to similar results between the first and second operations for exotropia of 15 to 20 PD [14].

Many studies have compared ULR and BLR in moderate-angle intermittent exotropia. Some authors reported equal effectiveness in cases of mild-to-moderate intermittent exotropia [11,15]. However, to the best of our knowledge, there has as yet been no investigation conducted to compare ULR and RR surgical outcomes for moderate-angle intermittent exotropia. In the present study, we compared surgical results of ULR and RR for intermittent exotropia of 20 to 25 PD.

## Methods

The medical records of 180 consecutive patients who had undergone RR or ULR for basic or pseudo-divergence excess-type intermittent exotropia of 20 to 25 PD by one surgeon (D.G.C.) with minimum 2-year follow-up periods postoperatively were retrospectively reviewed. Patients were excluded if they had a history of strabismus surgery, trauma, paralytic or restrictive exotropia, vertical incomitance, other ocular disease, or systemic abnormalities such as Down syndrome or cerebral palsy. Informed written consent for the surgical procedures was obtained from all of the patients or their parents. This study was approved by the Institutional Review Board of Hallym University Medical Center.

We noted preoperative characteristics including age at surgery, sex, mean angle of exodeviation at distance and near, stereopsis, best-corrected visual acuity (BCVA), refractive error, presence of lateral gaze incomitance, amblyopia and anisometropia, follow-up period, and type of surgery performed.

Sensory status was evaluated with the Titmus Stereo test (Stereo Optical Co., Inc., Chicago, IL, USA) and the Worth 4-Dot test at distance and near. Stereoacuity of 100 seconds of arc or better was defined as good stereopsis. Data from any patient who did not cooperate in test performance was excluded. Lateral gaze incomitance was defined as a change of 10 PD or more in the right or left gaze as compared with the primary position. Amblyopia was defined as a between-eye difference of 2 lines or more in visual acuity, and anisometropia was defined as a between-eye spherical or cylindrical difference of more than 1.50 diopters (D). The control of exodeviation was scaled as good, fair or poor [16]. Good control was defined if the fusion breaks only after cover testing at distance fixation and resumes rapidly without need for a blink or refixation; Fair and poor control was defined if the subject blinks or refixates to control the deviation after disruption with cover testing at distance fixation and the subject who breaks spontaneously without any form of fusion disruption, or who does not spontaneously regain alignment despite blink or refixation, respectively.

All of the patients underwent a complete ophthalmologic examination prior to surgery. Deviation was measured using the alternate prism cover test for distance (6 m) and near (33 cm) in all 9 positions of gaze using accommodative targets with their best optical correction preoperatively. A modified Krimsky method was used in examination of a few uncooperative patients. If the exodeviation at distance was larger than 10 PD compared with that at near, one eye of the patient was patched for 1 hour in order to eliminate fusional convergence, and the alternate prism cover test was repeated at near and at distance. Although the deviation was measured for both distance and near, it was the distance deviation that was considered in planning the amount of surgery. All of the surgeries were performed under general anesthesia using the standard limbal approach without any hangback or adjustable sutures. The selection of the surgical procedure was made by the operating surgeon, who had no preference for ULR or RR. The surgical dosages of each group were based on the angles of distance exodeviation listed in Table 1.

**Table 1:** Surgical table for intermittent exotropia

**PD**	**Unilateral LR recession (mm)**	**LR recession/MR resection (mm)**
20	9.0	5.0/4.0
21-24	9.5	5.5/4.5
25	10.0	6.0/5.0

PD = prism diopters, LR = lateral rectus, MR = medial rectus.

Postoperative alignment at distance was measured at postoperative 1 day, 1 week, 1, 3 and 6 months, 1 and 2 years, and at final follow-up. On every visit, the subjective diplopia in the primary position was recorded. Abnormalities in duction and version were also examined in determining the lateral gaze incomitance and abduction deficits.

Alternate full-time patching was prescribed if patients complained of diplopia or developed esodeviation postoperatively, and was continued until the diplopia or esodeviation was resolved. If the esodeviation at postoperative 2 months was still unresolved, cycloplegic refraction was performed and refractive errors were recorrected. If the esotropia persisted with alternate patching for 2 months, base-out Fresnel press-on prisms (3 M Press-On Optics™; 3 M Health Care, St Paul, Minnesota, USA) were prescribed. Reoperation was considered for the patients with consecutive esotropia of 20 PD or more for more than 6 months postoperatively, or nonacceptance of conservative treatment by a patient.

The main outcome measures were postoperative exodeviation angles at distance and final success rates. A surgical success was considered to be an alignment within 10 PD. An overcorrection was defined as more than 10 PD of esotropia, and undercorrection was defined as more than 10 PD of exotropia. The secondary outcome measures were changes of stereopsis as well as postoperative diplopia and lateral gaze incomitance.

The Mann-Whitney U test was used to compare the preoperative characteristics and preoperative and postoperative angles of deviation between the two groups. The Pearson chi-square test was used to compare the surgical success rates at each postoperative visit. All of the statistical analyses were performed with SPSS software, version 12.0 K (SPSS Inc., Chicago, IL, USA). A p value of < 0.05 was considered to be statistically significant.

## Results

In this retrospective study, ULR was performed on 82 patients and RR on 98 patients. The patients’ preoperative characteristics, which were not significantly different between the two groups, are presented in Table 2.

**Table 2:** Preoperative characteristics of ULR and RR group

	**ULR group**	**RR group**	***P *** **value**
	**(n = 82)**	**(n = 98)**	
Age at surgery	5.93 ± 2.35	6.76 ± 2.31	0.105^a^
Sex (male/female)	42/40	47/51	0.863^b^
Preoperative mean exodeviation (PD)			
at distance	22.07 ± 2.48	22.76 ± 2.49	0.069^a^
at near	22.01 ± 3.41	22.87 ± 4.12	0.102^a^
Fusion on worth-4-dots (n)			
at far	29 (35.4%)	38 (38.8%)	0.637^b^
at near	46 (56.1%)	51 (52.1%)	0.587^b^
Good stereopsis (≤100 seconds)	58/72 (80.6%)	65/83 (78.3%)	0.731^b^
BCVA			
OD, mean (range)	20/22 (20/50 ~ 20/20)	20/22 (20/50 ~ 20/20)	0.650^a^
OS, mean (range)	20/22 (20/50 ~ 20/20)	20/23 (20/100 ~ 20/20)	0.297^a^
Refractive error (D)			
OD, mean (range)	−0.18 (-6.50 ~ +6.00)	0.00 (-8.00 ~ +6.50)	0.405^a^
OS, mean (range)	−0.09 (-7.00 ~ +6.50)	0.00 (-8.50 ~ +6.50)	0.407^a^
Lateral gaze incomitance (n)	2 (2.4%)	5 (5.1%)	0.357^b^
Amblyopia (n)	10 (12.2%)	15 (15.3%)	0.548^b^
Anisometropia (n)	4 (4.9%)	7 (7.1%)	0.528^b^
Follow-up (months)	53.8 ± 26.4	52.5 ± 27.4	0.482^a^

BCVA = best-corrected visual acuity, D = diopters, ULR = unilateral lateral rectus recession, RR = unilateral recess-resect.

PD = prism diopters, n = number.

^a^Mann-Whitney *U*-test, ^b^Pearson chi-square test.

The mean angles of deviation at postoperative 1 day were -0.49 (esodeviation) ± 2.69 PD in the ULR group and -1.98 ± 4.11 PD in the RR group, which showed significant overcorrection in the RR group compared with the ULR group (p = 0.021, Mann-Whitney *U*-test) (Table 3). Subsequently, the deviations became more exotropic in the ULR group than in the RR group, with a statistical significance until postoperative 3 months (p < 0.05). However, from postoperative 6 months, there was no significant difference of deviation between the two groups; +7.98 ± 7.21 PD (exodeviation) in the ULR group and +8.12 ± 8.64 PD in the RR group, at the final follow-up (p = 0.872). And, the amount of exodrift in ULR and RR groups showed no significant difference (p > 0.05, T-test) (Table 4).

**Table 3:** Angle of deviation at distance preoperatively and during postoperative follow-up

	**ULR group**	**RR group**	***P *** **value**^**a**^
	**(n = 82)**	**(n = 98)**	
Preoperative deviation (PD)	+22.07 ± 2.48	+22.76 ± 2.49	0.069
Postoperative deviation (PD)			
1 day	-0.49 ± 2.69	-1.98 ± 4.11	0.021
1 week	+1.51 ± 2.34	+0.31 ± 2.56	0.042
1 month	+3.41 ± 3.98	+1.63 ± 2.68	0.035
3 months	+4.41 ± 4.44	+2.57 ± 3.52	0.047
6 months	+5.79 ± 5.17	+4.52 ± 4.79	0.712
1 year	+6.21 ± 5.26	+4.58 ± 5.98	0.174
2 years	+6.90 ± 5.79	+5.85 ± 6.79	0.334
Final follow-up	+7.98 ± 7.21	+8.12 ± 8.64	0.872

ULR = unilateral lateral rectus recession, RR = unilateral recess-resect, PD = prism diopters.

^a^Mann-Whitney *U*-test.

In the angle of deviation, the minus means esodeviation, and plus means exodeviation.

**Table 4:** The amount of exodrift in ULR and RR groups

	**ULR group**	**RR group**	***P *** **value**^**a**^
	**(n = 82)**	**(n = 98)**	
1 day to 1 months	3.90 ± 3.81	3.61 ± 4.27	0.635
1 months to 3 months	1.00 ± 2.93	0.98 ± 2.01	0.869
3 months to 6 months	1.37 ± 3.36	1.95 ± 3.06	0.235
6 months to 1 year	0.41 ± 1.34	0.90 ± 3.20	0.167
1 year to 2 years	0.68 ± 1.56	1.03 ± 3.36	0.364
2 years to last f/u	1.08 ± 2.40	1.66 ± 5.44	0.346

ULR = unilateral lateral rectus recession, RR = unilateral recess-resect.

^a^T-test.

Surgical success was achieved in 74 patients (90.2%) of 82 patients in the ULR group and 97 patients (99.0%) of 98 patients in the RR group at postoperative 1 month, and 69 (84.1%) in the ULR group and 94 (95.9%) in the RR group at postoperative 3 months, which showed better surgical results in RR group (p = 0.007, p = 0.007, respectively, Pearson chi-square test) (Table 5). However, there was no significant difference in the success rate at postoperative 6 months, 1 and 2 years (p = 0.171, p = 0.916, p = 0.659, respectively). A successful outcome at final follow-up was achieved in 50 patients (60.9%) in the ULR group and 55 (56.1%) in the RR group (p = 0.511).

**Table 5:** Surgical success rates in ULR and RR groups

	**ULR group**	**RR group**	***P *** **value**^**a**^
	**(n = 82)**	**(n = 98)**	
1 month	74 (90.2%)	97 (99.0%)	0.007
3 months	69 (84.1%)	94 (95.9%)	0.007
6 months	59 (72.0%)	79 (80.6%)	0.171
1 year	55 (67.1%)	65 (66.3%)	0.916
2 years	52 (63.4%)	59 (60.2%)	0.659
Final follow-up	50 (60.9%)	55 (56.1%)	0.511

Surgical success = alignment within 10 PD.

ULR = unilateral lateral rectus recession, RR = unilateral recess-resect.

^a^Pearson chi-square test.

No patient in either group was overcorrected at final follow-up postoperatively (Table 6). Undercorrection occurred in 32 patients (39.1%) in the ULR group and 43 (43.9%) in the RR group at final follow-up (p = 0.51, Pearson chi-square test). Reoperations were performed on 9 patients (10.9%) in the ULR group and 8 (8.2%) in the RR group during the follow-up period (p = 0.521).

**Table 6:** Surgical results at final follow-up postoperatively in ULR and RR groups

	**ULR group**	**RR group**	***P *** **value**^**a**^
	**(n = 82)**	**(n = 98)**	
Surgical success	50 (60.9%)	55 (56.1%)	0.511
Undercorrection	32 (39.1%)	43 (43.9%)	0.511
Overcorrection	0	0	-
Reoperation	9 (10.9%)	8 (8.2%)	0.521

ULR = unilateral lateral rectus recession, RR = unilateral recess-resect.

^a^Pearson chi-square test.

On preoperative examination, good stereopsis (≤100 seconds) was shown in 58 (80.6%) of 72 cooperative patients in the ULR group and 65 (78.3%) of 83 cooperative patients in the RR group (p = 0.731, Pearson chi-square test) (Table 2). Postoperatively, bad stereopsis (>100 seconds) was improved in 6 (42.8%) of 14 patients in ULR group and 10 (55.6%) of 18 in RR group. The improvement of stereopsis was not significantly different between the two groups (p = 0.722, Fisher’s exact test). Lateral gaze incomitance was presented in 5 ULR patients (6.1%) at postoperative 1 day, which lasted until postoperative 1 week in 3 patients (3.7%) and 1 month in 2 patients (2.4%), whereas none of RR patients had lateral gaze incomitance postoperatively. Eighteen ULR patients (22.0%) and 24 RR patients (24.5%) complained of diplopia at postoperative 1 day. Among them, only 8 ULR patients (9.8%) and 12 RR patients (12.2%) complained of diplopia at postoperative 1 week, and none after postoperative 1 month.

## Discussion

In this study, we compared the surgical results of the ULR and RR procedures for moderate-angle intermittent exotropia. The success rates for the two groups were similar, and decreased during the follow-up period in both. Disappointingly, surgical success at final follow-up (53.8 ± 26.4 months, 52.5 ± 27.4 months, respectively) was achieved in only 50 ULR patients (60.9%) and 55 RR patients (56.1%) (p = 0.511). In the study of Lee et al. [17], 62 patients with intermittent exotropia of less than 25 PD received ULR surgery of 8.5 to 9.5 mm. After 1 year of follow-up, 85.7% of the patients had an alignment within 10 PD, and no overcorrection or abduction limitation was found. Dadeya and Kamlesh [9] conducted a prospective study on 32 patients with intermittent exotropia of 25 to 30 PD who had undergone 8 mm ULR and were followed-up on for a minimum of 6 months. At 3 years follow-up, the success rate, defined as alignment within 5 PD, was 77.7%. Similarly, in the prospective study of Jeoung et al. [18], 55 of 66 patients (83.3%) with exotropia of 10 to 50 PD who received RR surgery had a satisfactory outcome, which is to say, within 10 PD at postoperative 6 months. Direct comparison of success rates among studies is not possible, owing to the different lengths of follow-up after surgery and the different criteria used for surgical success. Our current study included patients with intermittent exotropia of 20 to 25 PD, all of whom were followed-up on for more than 24 months, the mean follow-up periods being 53.8 ± 26.4 months and 52.5 ± 27.4 months for the two groups, respectively. Surgical success was defined as alignment within 10 PD.

Even though Leow et al. [19] reported that their success rate appeared to be unaffected by initial postoperative deviation, and Choi et al. [20] found that the advantage of initial overcorrection for exotropia disappeared after 5 years of follow-up. Many ophthalmologists still believe that early postoperative overcorrection is desirable for long-term stable functional results after exotropia surgery. Oh and Hwang [21] reported that the likelihood of a good postoperative surgical outcome was highest with an initial postoperative alignment of more than 10 PD of esotropia. In the present study, early overcorrection occurred at postoperative 1 day in both groups (mean esodeviation: -0.49 PD, ULR group; -1.98 PD, RR group). The mean deviations became more exotropic in the ULR group than in the RR group until 3 months postoperatively (p < 0.05). However, in both groups, the exodeviation tended to increase as the follow-up period became longer, and there was no significant difference between them from 6 months postoperatively (p > 0.05).

Lateral gaze incomitance is a worry for surgeons advocating ULR for moderate intermittent exotropia. Several studies have reported that lateral gaze incomitance was not observed after surgery on the unilateral rectus muscle for moderate-angle intermittent exotropia [13-15,17,22], whereas in some other investigations, it was verified only after ULR of more than 9 mm [8,9]. We defined the lateral incomitance as a change of 10 PD or more in the right or left gaze as compared with the primary position. In our study, recession up to 8-10 mm was performed, after which, at postoperative 1 day, lateral gaze incomitance was found in 5 patients. But, it decreased with time, so, no lateral gaze incomitance was observed in any of the patients from postoperative 2 months.

One-muscle surgery like ULR has the advantages of shortening the anesthesia time and decreasing the risks associated with surgery and tendency to bleed [6-8]. Moreover, ULR can lower the rate of overcorrection and resolve postoperative diplopia earlier than RR surgery [9-11]. Another advantage of one-muscle surgery like ULR is that other muscles are left untouched for repeat surgery [12]. Menon et al. [15] reported that ULR surgery appears to be as effective as BLR in cases of moderate intermittent extropia of 15 to 25 PD and, furthermore, that the likelihood of development of overcorrection and its related complications is less than with BLR. In our study, postoperative diplopia appeared only up to postoperative 1 week in both groups, and no overcorrection was found in either group.

There are some limitations to this study. First, it was a retrospective study for which the surgeon determined the surgical modality without specific selection policy; thus, selection bias could have occurred. Nonetheless, because the pertinent demographic data did not significantly differ between the two patient groups (Table 2), this study remains a useful comparative case series. Another drawback of this study was the fact that some patients having successful results did not return to the clinic, and some of the patients with unfavorable results were followed-up on longer than others. This might have resulted in a higher recurrence rate.

## Conclusions

ULR was as effective as unilateral RR in the treatment of moderate-angle intermittent exotropia of 20 to 25 PD, and it resolved postoperative esodeviation earlier. However, the results showed that the exo-drift tended to increase with longer follow-up periods in both groups. Proper management of patients with moderate-angle horizontal deviations remains a challenge for the strabismus surgeon. Future prospective and comparative studies with larger samples are required in order to confirm the effectiveness of unilateral rectus muscle surgery in cases of moderate-angle deviations and to determine the adequate amounts of unilateral rectus muscle surgery necessary to correct the strabismus without resultant lateral gaze incomitance or abduction limitation.

## Abbreviations

ULR: Is used for ‘unilateral lateral rectus recession’; RR: Is used for ‘unilateral recess-resect’; PD: Is used for ‘prism diopters’; BLR: Is used to abbreviate ‘bilateral lateral rectus recession’.

## Competing interests

The authors declare that they have no competing interests.

## Authors’ contributions

HJK contributed to study design and coordination, conducted the data analysis, and assisted in drafting and revising the manuscript. DK was involved in data acquisition, data interpretation, and critical revision of the manuscript. DGC helped to interpret the data and to draft and revise the manuscript. All authors have given final approval of the version to be published.

## Pre-publication history

The pre-publication history for this paper can be accessed here:

http://www.biomedcentral.com/1471-2415/14/46/prepub
